# A Model of Death Preparedness in Patients With Advanced Cancer: A Grounded Theory Study

**DOI:** 10.1111/jan.16669

**Published:** 2024-12-08

**Authors:** Xi Zhang, Tieying Zeng, Meizhen Zhao, Yisui Su, Xiaohong Liu, Ye Chen

**Affiliations:** ^1^ Department of Nursing Tongji Hospital, Tongji Medical College, Huazhong University of Science and Technology Wuhan China; ^2^ School of Nursing Tongji Medical College, Huazhong University of Science and Technology Wuhan China

**Keywords:** advanced cancer patients, core connotation, death preparedness, grounded theory, influencing factor, qualitative research

## Abstract

**Background:**

Death preparedness in patients with advanced cancer is an important prerequisite for improving the quality of death. However, there are insufficient studies on death preparedness in patients with advanced cancer, and the level of death preparedness needs to be further improved.

**Aim:**

To develop a model of death preparedness in patients with advanced cancer.

**Methods:**

A qualitative approach with grounded theory was used. Data were collected between February 2024 and July 2024 in the oncology wards of the two general hospitals in Wuhan. We recruited 12 patients, 11 family members, 16 nurses and 4 doctors for semistructured interviews. Data analysis included open coding, axial coding and selective coding. The study is reported using the COREQ checklist.

**Results:**

Death preparedness in patients with advanced cancer is a spiralling process whose core components include death awareness, emotional response, hospice programme and reflexive care, and multiple personal, interpersonal and social factors influence it.

**Conclusion:**

A model of death preparedness in advanced cancer patients was constructed through rooted theory, revealing its formation and change process. This model deepens the understanding of death preparedness and helps healthcare providers identify patients' preparedness status in advance to provide more targeted support and care. This personalised care enhances patients' quality of life and reduces the psychological burden on them and their families, achieving more comprehensive and humanised end‐of‐life care.

**Impact:**

To better understand patients' death preparedness, healthcare providers should focus on patients' cognitive, emotional, behavioural and social needs in the process of death preparation from a multifactorial perspective, and provide targeted support and assistance.

No Patient or Public Contributions were included in this paper.

## 1 Introduction

Currently, the incidence of cancer is rising rapidly worldwide. It is a serious threat to human health and life. According to the Global Cancer Statistics Report 2020 released by the International Agency for Research on Cancer (IARC) of the World Health Organization (World Health Organization 2024; Sung et al. 2021), there were 19.29 million new cancer cases and 9.96 million deaths worldwide in 2020. Among these, 4.57 million new cancer cases were reported in China, accounting for 23.7% of the global incidence, and there were 3 million deaths, which accounted for about 30% of the world's total cancer deaths (Cao et al. 2022). Malignant tumours have become one of the main causes of death in China, and it is expected that by 2040, the number of new cancer cases in China will reach 6.85 million (Cao et al. 2021; Zhou, Wang, and Zeng 2020). Shockingly, a proportion of cancer patients are already in the middle and late stages of the disease when they are diagnosed (Zeng et al. 2021). Despite high medical costs and extensive anticancer interventions, some cancer patients still face death within 1 year of diagnosis and endure prolonged and multiple torments at the end of life (Hagarty et al. 2020; Zeng et al. 2024).

In recent years, as our population ages and the incidence of cancer increases, it has become increasingly urgent and important to provide hospice care for patients with advanced cancer (Li et al. 2023; Stegmann et al. 2021). Additionally, with societal progress and improving living standards, people are gradually paying more attention to and pursuing the quality of life and the quality of death, making it imperative to enhance death preparedness for patients with advanced cancer (Mah et al. 2023). Death preparedness refers to an individual's preparedness to face death, which is an important prerequisite for improving the quality of death (McLeod‐Sordjan 2014). Chunlestskul et al. (2008) found that death preparedness benefits patients by enabling them to live fully and face death calmly. Steinhauser et al. (2008) demonstrated that enhancing patients' death preparedness effectively improves the quality of life for terminally ill patients while reducing anxiety and depression. Thus, for patients themselves, death preparedness can reduce death‐related anxiety and stress, promote a positive understanding of death, avoid unnecessary medical interventions and enhance quality of life at the end of life (Chan et al. 2006; McLeod‐Sordjan 2014). However, studies have shown that most patients with advanced cancer are unprepared for the reality of impending death, and despite receiving information about death, they still experience a sense of uncertainty (Ray et al. 2006). A survey of 383 patients with advanced cancer found that 17.1% were completely unprepared (Wen et al. 2021a).

Inadequate death preparedness in patients with advanced cancer can have a range of adverse consequences. Studies have found that when patients are unprepared for death, they often choose to escape their reality and are unwilling to confront their actual condition, leading to increased uncomfortable symptoms at the end of life (Huda, Shaw, and Chang 2022; Iskandar, Rochmawati, and Wiechula 2021). Simultaneously, these patients tend to experience a greater psychological burden and are less willing to communicate with their families and doctors, which exacerbates feelings of social alienation and isolation, ultimately affecting the quality and dignity of their lives (Iskandar, Rochmawati, and Wiechula 2021). For family members, inadequate death preparedness in patients with advanced cancer can result in poor communication, making it difficult for them to understand the patient's true needs. This can lead to biased decision‐making and prolonged grief responses (Hebert et al. 2006; Steinhauser et al. 2001). On a societal level, inadequate death preparedness may lead to an over‐reliance on high‐cost medical interventions, resulting in inefficient allocation of healthcare resources (Liu and van Schalkwyk 2019). In recent years, death preparedness in patients with advanced cancer has gradually attracted significant attention from scholars, and related research is on the rise. However, the current theoretical model of death preparedness for these patients remains underexplored. There is still a lack of a unified definition regarding the concept and connotation of death preparedness, and its specific content has not been clearly delineated (McLeod‐Sordjan 2014; Wen et al. 2021b). Additionally, research on factors affecting death preparedness is still insufficient, with most studies focusing on quantitative analyses that fail to fully consider patients' subjective experiences and individual differences. A more comprehensive and in‐depth exploration is urgently needed to enhance our understanding of death preparedness in patients with advanced cancer.

In summary, death preparedness plays a crucial role in terms of quality of life and quality of death in patients with advanced cancer (Chan et al. 2006; McLeod‐Sordjan 2014); however, there is still a significant knowledge gap in understanding death preparedness in patients with advanced cancer, especially from a qualitative perspective. Furthermore, death preparedness as a complex phenomenon is influenced by cultural factors, and a comprehensive theoretical model has yet to be developed to explain this phenomenon. In view of this, from the perspective of integrating theory and practice, this study adopts a grounded theory research methodology suitable for conducting theory building to develop a model of death preparedness in patients with advanced cancer. This will help to advance the development and measurement of death preparedness in patients with advanced cancer and facilitate its application in nursing practice.

## 2 The Aims of the Study

The research question of this study focused on exploring death preparedness in patients with advanced cancer. The main aim was to construct a model of death preparedness in patients with advanced cancer.

## 3 Methods

### 3.1 Study Design

We used the grounded theory research method developed by Strauss and Corbin (2008). Grounded theory is a method for exploring phenomena when little is known about the field of study (Birks and Mills 2011). This method helps to provide new insights into phenomena and develop theories that describe them, offering insights and theoretical support for academic research and practice (Moore 2009). Corbin and Strauss' (1990) grounded theory, which is based on a pragmatic philosophical perspective, provides researchers with a way to better understand and explain death preparedness in patients with advanced cancer. In this study, we applied grounded theory to construct a model of death preparedness in advanced cancer patients. The Consolidated Criteria for Reporting Qualitative Research (COREQ) checklist (Tong, Sainsbury, and Craig 2007) was used to guide this study, and it is detailed in Data S1.

### 3.2 Participants and Sampling

To comprehensively explore death preparedness in patients with advanced cancer, this study was designed from a multifaceted perspective, including participants such as patients, their families and healthcare providers who have close, direct interactions with these patients.

A combination of purposive and theoretical sampling was used to select eligible participants. To better explore death preparedness in patients with advanced cancer, this study was designed as a two‐phase study. The first phase, the exploratory phase, aimed to comprehensively understand participants' perceptions of death preparedness and refine the interview guide for subsequent stages. The second phase was focused, where participants were initially sampled purposively and then theoretically sampled to inform further sampling. Additionally, consideration of various factors, such as age and education level, ensured the development of a robust model, which concluded sampling when theoretical saturation was reached. Participants' inclusion and exclusion criteria were the same for both stages, with specific criteria detailed in Table 2.

### 3.3 Data Collection

Data were collected between February 2024 and July 2024 in the oncology wards of the two general hospitals in Wuhan. Data were collected by the primary researcher, a nursing PhD candidate, with the systematic study of qualitative research methods and practice in the field. All patients and patient's family members who met the inclusion criteria were given the choice of face‐to‐face, one‐on‐one interviews in a single ward or dedicated conference room; for healthcare providers who met the inclusion criteria, they were given the choice of either face‐to‐face interviews or Tencent video conferencing for online interviews to better co‐operate with their work. Prior to data collection, the relationship between the researcher and the participants had not been established; however, the purpose of the study was discussed at the beginning of the interview. To thank the participants, we prepared gift packs that included wet wipes, hand cream and other items.

Between 26 February 2024 and 20 March 2024, the first data collection phase was completed, focusing on participants' perceptions of death preparedness in patients with advanced cancer. Randomised probing questions were used to examine participants' responses in more depth. Seven different study participants (three patients, two family members, one nurse and one doctor) were recruited for preinterviews, which were preliminarily analysed and adapted to determine the final version of the interview outline (see Table 1).

**TABLE 1:** Overview of questions used in the interview guide.

Role	Interview questions
Patients	What have been your psychological changes from the beginning of your illness until now? Have you ever considered death? What do you think death preparedness means at the end of life? What specific aspects can be included? Please give examples. What do you think are the influencing factors that affect your death preparedness? What do you think is the best death preparedness? Is there anything else you would like to tell us?
Family members	What have been your psychological changes since the onset of the patient's illness? Have you ever considered the patient's death? What do you think death preparedness means at the end of life? What specific aspects can be included? Please give examples. What do you think are the influencing factors that affect patient's death preparedness? What do you think is the best death preparedness? Is there anything else you would like to tell us?
Healthcare providers	What do you think death preparedness means at the end of life? What specific aspects can be included? Please give examples. What do you think are the influencing factors that affect patient's death preparedness? What do you think is the best death preparedness? What do you think is the significance of death preparedness for people with advanced cancer? Is there anything else you would like to tell us?

**TABLE 2:** Inclusion and exclusion criteria for study participants.

Participants	Inclusion criteria	Exclusion criteria
Patients	(1) cancer diagnosed histologically or cytologically as malignant and at stage III or IV according to the TNM (Edge and Compton 2010) tumour staging system; (2) age ≥ 18 years; (3) aware of their condition; (4) clear consciousness and able to communicate normally; (5) informed consent and voluntary participation in the study	Patients with a history of mental illness and cognitive impairment
Family members	(1) immediate family members (spouse, children, parent) of the patients; (2) age ≥ 18 years; (3) main caregiver of the patient and clear awareness; (4) informed consent and voluntary participation in the study	People with mental illness and cognitive impairment
Healthcare providers	(1) possession of a licence to practise; (2) experience of direct contact with patients with advanced cancer and (3) informed consent and voluntary participation in the study	Healthcare providers with a history of mental illness and those who had studied, practised or trained in the survey hospitals

**TABLE 3:** Demographic characteristics of participants and their roles.

Characteristics	Frequency	Percentage
*Patients* (*n* = *12*)		
Gender		
Female	7	58.33%
Male	5	41.67%
Age		
Mean	44	
Min–Max	27–76	
Education		
Junior high school and lower	6	50%
Senior high school	4	33.33%
University and higher	2	16.67%
Household per capita monthly income		
< ¥3000	5	41.67%
¥3000–6000	2	16.67%
¥6000–9000	2	16.67%
>¥9000	3	19.99%
Residence		
Urban	7	58.33%
Rural	5	41.67%
Cancer diagnosis		
Lung cancer	2	16.67%
Liver cancer	1	8.33%
Gastric cancer	2	16.67%
Colorectal cancer	1	8.33%
Breast cancer	2	16.67%
Others^a^	4	33.33%
*Family members* (*n* = *11*)		
Gender		
Female	7	63.64%
Male	4	36.36%
Age		
Mean	41	
Min–Max	26–68	
Education		
Junior high school and lower	6	54.55%
Senior high school	3	27.27%
University and higher	2	18.18%
Relationship with patients		
Spouse	4	36.36%
Children	3	27.28%
Parent	4	36.36%
*Healthcare providers* (*n* = *20*)		
Gender		
Female	16	80%
Male	4	20%
Age		
Mean	36	
Min–Max	28–51	
Education		
Bachelor's Degree	10	50%
Master's Degree	6	30%
Doctorate	4	20%
Occupation		
Nurses	16	80%
Doctors	4	20%
Working years		
< 10	5	25%
10–15	9	45%
> 15	6	30%

^a^ Pancreatic cancer 1; oesophageal cancer 1; lymphatic cancer 1; cervical cancer 1.

**TABLE 4:** Coding process of death preparedness in patients with advanced cancer model.

Selective coding	Axial coding	Open coding	
		Category	Concept
The core connotations of death preparedness in advanced cancer patients	Death awareness	Symptom control	Pain
			Nausea and vomiting
			Bloating
			Fatigue
			Breathlessness
			Sleep disturbances
		Prognostic awareness	Survival time
			Chance of recovery
			Disease progression
			Quality of life
		Death consciousness	Self‐perception
			Mortality Salience
			Death reflection
	Emotional response	Fear of death	Escape from reality
			Fear of being forgotten
			The cycle of life and death
		Pursuit of life meaning	Self‐value discovery
			Self‐empowerment
			Meaning of existence
		Self‐acceptance	Reconciliation with others
			Self‐identification
			Death acceptance
	Hospice programme	Living will	Advance planning
			Participate in decision‐making
			Life autonomous
			Treatment options
		Making arrangements	Cemetery selection
			Funeral planning
			Social work handover
		Financial arrangements	Medical expenses
			Living expenses
			Grafting of wills
	Reflexive care	Relationship sublimation	Valuing family and friends
			Being grateful for companionship
			Cherishing the years
		Material contributions	Bequests of property
			Funding of charities
			Organ donations
		Spiritual inheritance	Leaving behind life experience
			Transmitting love and hope
			Demonstrate life and death attitude
The influencing factors of death preparedness in advanced cancer patients	Personal factors	Demographic characteristic	Gender
			Age
			Economic level
			Education
			Death‐related experience
			Beliefs
		Disease factors	Diagnosis
			Metastatic status
			Staging
			Pain level
		Sense of Meaning in Life	Need for Meaning of Life
			Experience of Meaning of Life
		Patient dignity	Symptom distress
			Existential distress
			Dependency
			Peace of mind
			Social support
		Coping style	Confrontation
			Avoidance
			Acceptance resignation
	Interpersonal factor	Social support	Family support
			Friend support
			Other support
		Communication situation	Family communication
			Healthcare communication
		Family care	Adaptation
			Partnership
			Growth
			Affection
			Resolve
		Caregiver preparedness	Physical care needs
			Emotional needs
			Caregiving stress
		Healthcare personnel preparedness	Needs and services
			Care situation
			Emergency management
	Social factor	Policy support	Insurance
			Financial subsidies
			Death education
		Care resources	Physical care
			Medical care
			Psychological support
		Information supply	Treatment modalities
			Drug information
			Treatment outcomes
			Disease progression
			End‐of‐life preparedness knowledge
			Access avenues

Between 22 March 2024 and 8 July 2024, the second data collection phase was completed. Interviews were audio‐recorded after informed consent was obtained from the participants. To ensure the interviews were as effective as possible, a quiet environment was chosen to minimise disturbances. The interviews generally lasted 45–60 min. They were terminated at any point during the process if the participants experienced any discomfort, and free psychological counselling was provided to assist them. The researcher maintained a neutral attitude and encouraged participants to share their inner experiences while observing their expressions and movements, recorded through field notes. In addition, thoughts about the interviews were documented in the form of memos. While adhering to the interview outline, the order of questioning was adjusted based on participants' responses to maintain flexibility and avoid interrupting their contemplation. Theoretical adequacy was achieved in 40 interviews during this phase. Three additional interviews were conducted to ensure no new concepts or categories emerged. The study included a total of 43 research participants.

### 3.4 Data Analysis

Data collection and analysis took place simultaneously throughout the study. All recordings were transcribed verbatim within 24 h of interview completion and subsequently imported into Nvivo11.0 software for data analysis. The data analysis process strictly adhered to the methodological framework described by Strauss and Corbin (Strauss and Corbin 2008), including open coding, axial coding and selective coding, and continued until theoretical saturation was achieved. Memos were used to record procedural aspects of the analysis process and any insights that arose, including the addition of sentiment and text to reflect the meaning of words and tone. Prior to coding, two researchers reviewed the transcribed text multiple times to ensure a comprehensive grasp of the data. Constant comparisons were used when analysing the data to avoid bias and achieve greater accuracy during the coding process.

In the open coding phase, textual information was reviewed iteratively and meaningful units were extracted to form codes. Two strategies were used, questioning the data and constant comparison. After careful review, a total of 89 codes were generated through open coding and subsequently categorised into 25 initial categories.

In the axial coding stage, the open codes were further processed and analysed to explore the logical relationships between the open codes and to form categories from the processes by which the codes are interrelated, in order to group similar codes with each other and to reduce the number of categories. The categories were merged and formed into axial categories. After axial coding, the 25 initial categories were merged into seven subcategories.

**FIGURE 1:**
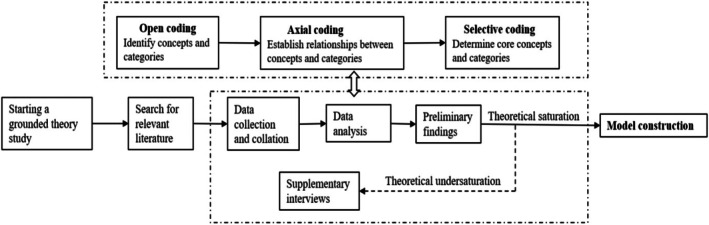
Data collection and analysis process.

**FIGURE 2:**
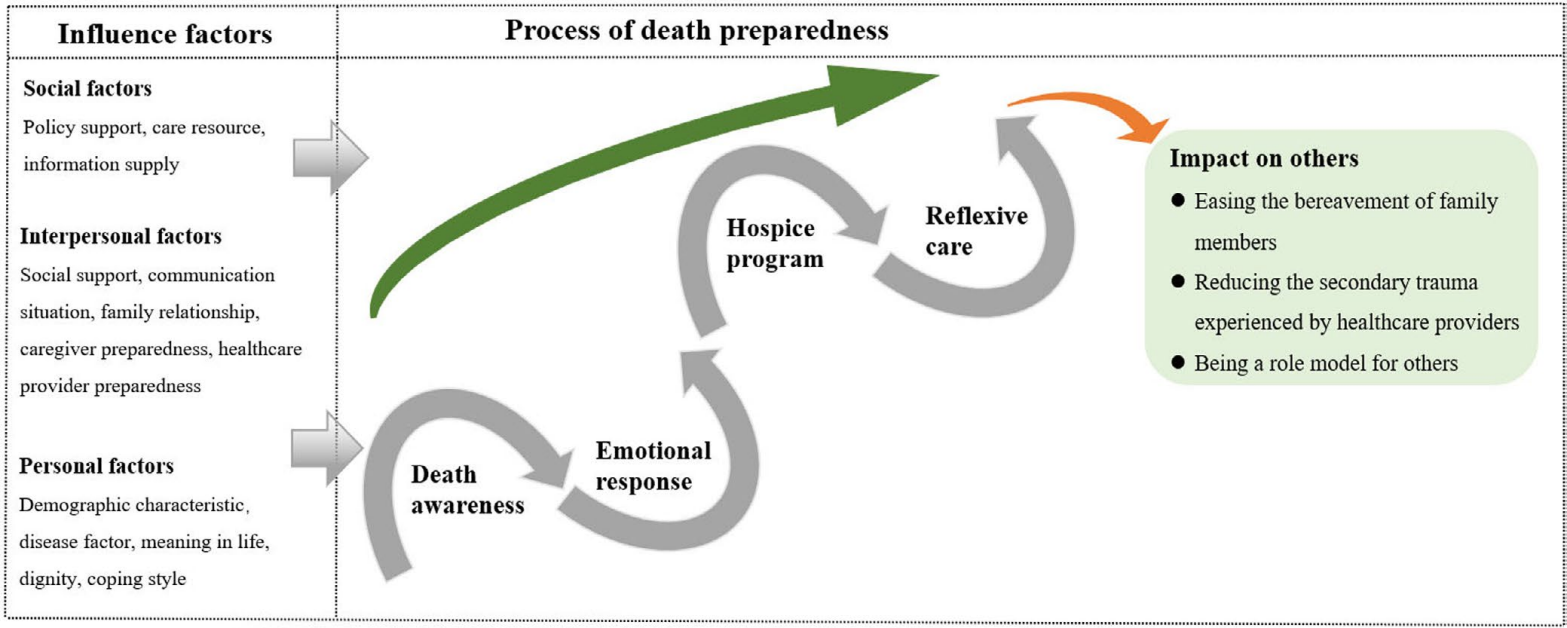
A model of death preparedness in patients with advanced cancer.

The selective coding phase emphasised the identification of a core category that systematically linked other categories. Two main categories were derived from a constant comparison of all categories and subcategories: the core connotations of death preparedness in advanced cancer patients, and the influencing factors of death preparedness in advanced cancer patients. The concept of ‘self‐reconciliation in the struggle between life and death’ emerged as the core category that dominated the main categories. Figure 1 illustrates the process of data collection and analysis throughout the study. In addition, a model was constructed to elucidate the phenomenon (see Figure 2).

Throughout the data analysis process, the two researchers coded the data separately and then continually compared the results until they reached an agreement on the codes. Personal opinions were set aside during the analysis process. Additionally, the researchers maintained a detailed record of the analysis steps while regularly reflecting on their opinions and potential biases. In the case of disagreement, the opinion of the corresponding author was sought to make a final decision.

### 3.5 Rigour and Trustworthiness

This study used credibility, reliability, confirmability and transferability to ensure rigour (Johnson, Adkins, and Chauvin 2020). Credibility was established by strictly following Strauss and Corbin's grounded theory methodology. To enhance reliability, the researcher engaged in a systematic qualitative research study and maintained complete neutrality during the interview process. To ensure confirmability, the researcher recorded ideas and reflections through memos and, after obtaining initial codes, discussed them with researchers who had relevant experience. Finally, the researcher described all factors involved, including participant selection criteria, data collection and analysis processes, and research limitations to achieve transferability.

### 3.6 Ethical Considerations

The study met the requirements of the Declaration of Helsinki and received ethics approval from the Ethics Committee of Tongji Hospital, Tongji Medical College, Huazhong University of Science and Technology (TJ‐IRB202404102). We explained the purpose of the study in detail to the participants and obtained their written consent before the formal interview. Participants could terminate the interview at any time and refrain from discussing uncomfortable topics. Identifying information about anonymous and confidential participants is kept secure (e.g., patient P1, P2; family members F1, F2; healthcare providers N1, N2). For this study, all original recordings and texts are saved using cryptographic calculations to ensure confidentiality.

## 4 Results

A total of 12 patients, 11 family members, 16 nurses and 4 doctors were recruited for formal interviews. The demographic characteristics of the participants are shown in Table 3.

The model of death preparedness in patients with advanced cancer was the result of multilevel coding and constant comparison. Specific coding results are shown in Table 4. The model of death preparedness in patients with advanced cancer is shown in Figure 2.

### 4.1 Overview of the Model of Death Preparedness in Patients With Advanced Cancer

The model of death preparedness in advanced cancer patients (Figure 2): self‐reconciliation in the struggle between life and death in the process of death preparedness in patients with advanced cancer. This model describes the core connotation of death preparedness and the influencing factors in its formation process.

Within this theoretical framework, the process of death preparation in advanced cancer patients is a process of spiralling development. In essence, this process emphasises self‐reconciliation in the life and death struggle during the death preparedness process. The process of death preparedness consists of four main stages: death awareness (cognitive), emotional response (emotional), hospice program (behavioural) and reflexive care (social). At each stage, the patient goes through a process of self‐struggle, similar to a ‘U’ shape, and slowly progresses to the next stage. It is important to note that each stage may overlap, depending on the individual situation.

In the process of death preparation, various factors play a role, categorised into personal, interpersonal and social influences. Personal internal factors encompass demographic characteristics, disease factors, life meaning, dignity and coping styles. Interpersonal external factors involve social support, communication, family relationships, caregiver preparedness and healthcare provider preparedness. Social factors include policy support, care resources and information supply. The complex interplay of these factors collectively shapes the model of death preparedness.

### 4.2 The Influencing Factors of Death Preparedness in Advanced Cancer Patients

This category illuminates that advanced cancer patients' death preparedness is influenced by a combination of multiple factors, including personal, interpersonal and social factors, which interact and influence each other to determine the degree of preparedness in the face of death.

#### 4.2.1 Personal Factors

Interviews revealed that different genders tend to exhibit different psychological and emotional responses when faced with death preparedness. Older patients were found to be more accepting of the reality of death. Additionally, varying literacy levels also affect patients' attitudes towards death preparedness.

N1: ‘Among the patients in my care, I feel that female, highly educated patients are generally more willing to discuss death and prepare for it in advance; male patients tend to be more likely to adopt an avoidant attitude toward impending death’.

P3: ‘I'm getting older, and I'm not afraid of dying……. I just want to prepare for death in advance while I'm still alive, so that I can reduce the burden on my children’.

In addition, the economic level and having experienced the death of a loved one were important factors influencing the level of death preparedness. We also observed that attitudes towards death preparedness can differ based on beliefs.

P10: ‘My brother passed away from esophageal cancer a few years ago; he had surgery, which cost a lot of money. I don't have enough money to support my ongoing treatment, and I'm now slowly starting to prepare for death’.

P8: ‘As a Buddhist, I believe life and death are part of a continuous cycle. Death is not the end of life, so I choose to prepare for it in advance’.

#### 4.2.2 Interpersonal Factors

Some patients reported that social support can help with support and comfort in the face of difficulties and more strength to face death; communication with family members, healthcare professionals and family care can also influence death preparedness.

P2: ‘Although I have advanced pancreatic cancer, I am truly blessed by the care and support. I feel surrounded by love……I am no longer afraid of death and plan to say “goodbye” to my family in advance’.

F6: ‘Upon learning that my husband's illness was incurable and his time was limited, we held a family meeting and communicated fully with the doctors. We wanted to do as much as we could for him to minimize the regrets of his life’.

We found that caregiver preparedness, as well as healthcare provider preparedness, also affects patients' death preparedness to some degree, affecting their end‐of‐life experience and psychological state.

N14: ‘When we clearly inform the patient that further treatment is no longer possible, even if they cannot accept it at first, they will gradually begin to face reality and start making death preparedness’.

P1: ‘My illness has reached its terminal stage… As the sole child in my family, I can clearly sense that my parents are completely unprepared for the news of my critical illness… I really love them, and I don't want to die’.

#### 4.2.3 Social Factors

We found that patients consciously seek information on treatment options and hospital policies during hospitalisation. In addition, patients need specialised nursing care and psychological support, all of which can influence their attitudes towards preparing for death.

P10: ‘Sometimes, when I contemplate the untreatable nature of my disease, I am so sad that I even want to commit suicide …… My charge nurse often enlightens me and encourages me to cherish my remaining time and strive to reconcile my regrets’.

F4: ‘The medical staff were very professional and attentive in their care, and they made us feel safe…… We have a major medical allowance …… Being in hospital, even if it did come to death, would have made my daughter suffer less’.

In addition, information supply also affects the death preparedness in patients with advanced cancer. Some patients said that being informed about their disease and treatment promptly could help them prepare for it in advance.

P7: ‘My family members kept information about the disease from me…… I want them to be honest with me so I can be prepared for my death’.

P9: ‘I hope the doctor can tell me directly how long I can live so I can plan the rest of my time and make some preparations for death, such as arranging for money and the house…… I want to spend the remaining time accompanied by my children’.

### 4.3 The Core Connotations of Death Preparedness in Advanced Cancer Patients

Death preparedness in advanced cancer patients is an evolving process that includes cognitive, emotional, behavioural and social aspects, and this category includes four subcategories.

#### 4.3.1 Death Awareness

As advanced cancer progresses, patients often become more acutely aware of its symptoms. Participants reported being well aware of the treatment's side effects and how to manage potential symptoms. For many patients and their loved ones, the realisation that treatment can only slow disease progression, not offer a complete cure, presents a complex and difficult reality.

P1: ‘My body pains are getting worse now. I have mouth ulcers, vomiting, fatigue, etc., and I can feel that the cancer has spread to many parts of my body’.

F1: ‘His current treatment has no effect at all, and it is estimated that continuing the treatment is not possible …… can only be accompanied by one more day’.

Patients tend to have an intuitive awareness when faced with death. When patients are confronted with a serious illness or other situation that could lead to death, they tend to feel the presence of death more directly.

P2: ‘These days, I often dream of my grandmother; she has been dead for five years, and I will soon be able to accompany her’.

P7: ‘My family member asks me some wishes… Probably don't want me to have any regrets in the world… I feel like I'm at the end of my life’.

#### 4.3.2 Emotional Response

When people with advanced cancer become aware of death, they experience a range of emotional reactions. These emotional responses are often complex and mixed, with each person experiencing and coping uniquely with death. Some patients expressed fear and trepidation about the unknown that would follow death.

P3: ‘There will be nothing left of me when I die; my children are still so young…… they might not remember me when they grow up. I am truly terrified and wish to live for a few more years’.

P6: ‘Although I understand that everyone must face death, I still can't bring myself to accept it. I also can't fathom the suffocating sensation of nearing death’.

Participants expressed a desire to find and re‐examine what is meaningful and valuable in their lives and to reflect on their purpose and value in the world. Some patients said they accepted their past experiences, including their regrets and imperfections, while also recognising the achievements and meaning they have created.

P8: ‘I am no longer overly pursuing the right and wrong of previous things, that's in the past…… Anything that happens has meaning; I want to try to fulfill my value in life’!

F7: ‘Seeing my husband sick, I can feel the impermanence of life; it's better to cherish the present. I've been thinking a lot about how some things must be done before it's too late to avoid regrets…… Find your goals sooner rather than later’.

#### 4.3.3 Hospice Programme

As patients emotionally accept death, their behaviour and decisions may change significantly, and the end‐of‐life treatment choices they say they would consider include measures to discontinue treatment, advance directives, and so on.

P1: ‘I want to be able to leave the world in peace and with dignity when I die…… I do not want to sacrifice my basic dignity just to prolong life’.

P5: ‘When I reach the end of my life, I don't want any more invasive intubation or CPR……I want to go home and die there’.

To ease the burden on their loved ones and honour their wishes, patients often make arrangements for postdeath matters well in advance, including cemetery choices, funeral or memorial services and the transfer of responsibilities. Many patients also report examining medical care options and actively engaging in financial planning to safeguard themselves and their families.

P2: ‘When I die, I don't want my funeral to be lavish; I want it to be a simple ceremony. I have some savings, which I plan to leave to my kids’.

P11: ‘I am terminally ill, and I don't want to spend more money on surgery. I want to leave this money to my family. Much of my work is currently unscheduled.… I need someone to take over my work as soon as possible’.

#### 4.3.4 Reflexive Care

Patients said they would value their relationships with friends and relatives even more and recognise the preciousness of family and friendship; they would also express gratitude to everyone who has accompanied them and cherish the remaining time.

P6: ‘The healthcare providers and my family cared for me wholeheartedly…… I also wanted to show them some care in return’.

P9: ‘I now slowly realize the joy of being with my loved ones and cherish these moments. I want to spend more time with them in my limited life’.

Some patients considered giving back to society through material contributions, including bequeathing property, funding social welfare programmes or even being willing to donate organs. In addition, patients expressed a desire to leave behind their life experiences and wisdom in the form of text, audio and video.

N17: ‘Some patients would ask me about organ donation, expressing a desire to help someone in need. They want to help others live longer’.

P5: ‘I've remained strong since falling ill and have never wanted to back down from any challenge…… I would like to donate some of my wealth to help the poor in remote areas’.

## 5 Discussion

This study developed a multiperspective model of death preparedness in advanced cancer patients through interviews with patients, their families and healthcare providers. By integrating data, the study revealed the core concepts and key factors influencing death preparedness in advanced cancer patients. Unlike previous studies (Mack et al. 2008; McLeod‐Sordjan 2023), this study employed grounded theory to construct a model of death preparedness in patients with advanced cancer and in‐depth explored the formation process of death preparedness. The study's originality and utility are emphasised by providing new insights and conceptual developments. These findings and insights have important implications for future practice, education and policy development in death preparedness care for patients with advanced cancer.

Patient preparation for death is a process of self‐struggle, and self‐reconciliation is achieved through constant adjustment (Steinhauser et al. 2000; Wen et al. 2021a). Death preparedness in patients with advanced cancer is a spiralling process (Tang et al. 2019), with key core concepts including death awareness (cognitive), emotional response (emotional), hospice programme (behavioural) and reflexive care (social). In the process of death preparedness, patients with advanced cancer undergo a transition involving behavioural and social preparation in the awareness and acceptance of impending death (Vaidimarsdóttir et al. 2007; Wen et al. 2021b), which is consistent with the findings of Hebert et al. (2006). Different patients have different emotional responses to death, and unsurprisingly, there is a common fear of death in patients with advanced cancer (Vehling et al. 2017). For the patient, this fear usually stems from anxiety about the unknown and the perception of a diminishing sense of control over their lives (Alifrangis et al. 2011). Some patients also reflect on the meaning of life and slowly begin to self‐accept. Research has found that advanced cancer patients tend to make advanced hospice programmes at the end of life, including advance directives and financial and affairs arrangements (Pedrosa et al. 2023; Goswami 2023), which is highly consistent with our findings.

In addition, we discovered an interesting phenomenon: many patients at the end stage of the disease, in addition to worrying about their disease, think more about their responsibility to others, their families and society (Filiberti et al. 2001; Fischer and Seibaek 2021). In our traditional culture, when facing death, patients often pay more attention to their roles and responsibilities, hoping to protect and support their families in various ways to ensure that family members can continue to live a good life (Kang et al. 2015; Li, et al. 2023; Liu and van Schalkwyk 2019); It also pays attention to the inheritance of filial piety and the continuation and completion of social roles. They want to give reflexive care to others while receiving care from others (Wang and Liu 2019). This reflexive care reflects the patient's inner transformation in the face of death and demonstrates a sense of social responsibility (Huang et al. 2022; Kang et al. 2015). It can be part of patients' quest for inner peace and reconciliation at the end of their lives while also helping them leave a positive legacy on a personal level.

The model of death preparedness in patients with advanced cancer reveals that a complex multidimensional environment influences the formation of death preparedness in patients with advanced cancer. Personal, interpersonal and social factors affect patients' death preparedness to varying degrees (Hebert et al. 2006; McLeod‐Sordjan 2014; Prigerson and Maciejewski 2008; Steinhauser et al. 2000). Individual factors are determinants of death preparedness, including demographic characteristics such as gender, age, economic level, education, death‐related experiences and beliefs (Chan and Yau 2009; Gott et al. 2008; Wen et al. 2023). In addition, many patients reported being influenced by disease factors, sense of meaning in life, dignity and coping styles. The data presented here are primarily supported by previous researchers (Thompson et al. 2009; Wen et al. 2023), but provide additional findings for this area of research. Patients are affected not only by personal aspects but also by interpersonal factors because they are unlikely to live independently and are often influenced and interacted with by those around them (Hebert et al. 2006, 2009; Wen et al. 2022a). Interpersonal factors include social support, communication, family relationships and caregiver and healthcare provider preparedness (Wentlandt et al. 2012; Mah et al. 2020; Tang et al. 2020). The finding of these factors is crucial to understand and deal with the situation of the patient, for whom we need to give adequate social support to face the death better. Good communication and harmonious family relationships can help us understand our patients better and help them prepare for death (Hebert et al. 2009).

In addition, the preparedness of caregivers and healthcare providers is crucial (Tang et al. 2021; Wen et al. 2022b), as their expertise, understanding and care can directly affect the overall psychological state of the patient and enable the patient to receive adequate care resources (Alvariza et al. 2020). Contrary to the common belief that patients with advanced cancer cannot be informed about disease progression and preparation for death (Shahidi 2010; Sutar and Chaudhary 2022). Participants in this study expressed a desire for knowledge related to disease progression and preparation for death. At the same time, they reported that relevant policy support would also impact their death preparedness. Therefore, adequate policy support, care resources and information provision are integral to death preparedness in patients with advanced cancer. In summary, through in‐depth understanding and integrated consideration of personal, interpersonal and social factors in patients with advanced cancer, healthcare professionals can provide more comprehensive and effective clinical care to help patients with advanced cancer receive more support and care when facing death preparedness and to improve the level of death preparedness in patients with advanced cancer.

### 5.1 Impact

The theoretical model of death preparedness in advanced cancer patients developed in our study provides new understandings and perspectives for caring for such patients. By focusing on the cognitive, emotional, behavioural and social needs and influencing factors in the process of death preparedness, healthcare providers can gain a more comprehensive understanding of patients' psychological states and individualised needs, thereby providing more effective and compassionate care and support. Cross‐cultural validation studies and pilot studies are recommended to apply the findings to different populations, and a variety of research methods, including mixed methods, empirical studies and intervention studies, should be used to explore and refine the model further. In addition, death preparedness should be incorporated into nursing and medical education to enhance the awareness and skills of professionals to serve patients with advanced cancer better.

### 5.2 Limitations and Strengths

This study employed grounded theory to deeply explore the process of death preparedness in advanced cancer patients and develop a model explaining this phenomenon. Moreover, it delved into the core connotations of death preparedness in advanced cancer patients and identified the multifaceted influencing factors, thereby complementing the findings of previous studies.

However, this study has limitations. It was conducted within Chinese culture, and further field tests are needed to apply the findings to other cultural contexts. Additionally, this study is a theoretical construct lacking empirical evidence, necessitating continuous revision and refinement of the research design in future quantitative studies.

## 6 Conclusion

This study provides a theoretical model of death preparedness in advanced cancer patients within the Chinese cultural context. The core concepts of this model elucidate the processes involved in death preparedness, offering crucial insights for healthcare providers aiming to understand and support patients and their families during this challenging time. The study's findings indicate that death preparedness in advanced cancer patients is shaped by a multitude of factors that healthcare providers must consider. Building on these findings, this study further elaborates and develops the concept of death preparedness in advanced cancer patients, offering a theoretical framework for future research, measurement and practical application.

## Author Contributions

Xi Zhang contributed to writing – original draft, investigation, methodology, software data curation and formal analysis. Tieying Zeng was involved in supervision, funding acquisition and formal analysis. Meizhen Zhao participated in the investigation, data curation and project administration. Yisui Su and Xiaohong Liu contributed to the writing – revising draft. Ye Chen contributed to conceptualisation, methodology, formal analysis and writing – revising draft.

## Conflicts of Interest

The authors declare no conflicts of interest.

## Peer Review

The peer review history for this article is available at https://www.webofscience.com/api/gateway/wos/peer‐review/10.1111/jan.16669.

## Supporting information


Data S1.

## Data Availability

The datasets collected and analysed in this study are not available to the public due to ethical constraints to protect the anonymity of participants. The data that support the findings of this study are available from the corresponding author upon reasonable request.
